# Assessment of causal link between psychological factors and symptom exacerbation in inflammatory bowel disease: a systematic review utilising Bradford Hill criteria and meta-analysis of prospective cohort studies

**DOI:** 10.1186/s13643-020-01426-2

**Published:** 2020-08-01

**Authors:** Mariyana Schoultz, Michelle Beattie, Trish Gorely, Janni Leung

**Affiliations:** 1 School of Health and Society, University of Salford, Manchester, UK; 2 Centre for Health Sciences, University of Highlands and Islands, Inverness, Scotland, UK; 3 Faculty of Health and Behavioural Sciences, The University of Queensland, Brisbane, Australia

**Keywords:** Inflammatory bowel disease, Crohn’s disease, Ulcerative colitis, Psychological factors, Symptom exacerbation, Systematic review, Meta-analysis

## Abstract

**Background:**

Psychological stress is a prevalent factor in inflammatory bowel disease (IBD) with detrimental effects on patients’ quality of life and possibly disease course. Although the aetiology of symptom exacerbation in IBD has been explored, determining any causation between psychological stress and symptom worsening remains challenging and requires a methodologically rigorous approach.

**Aim:**

The aim of this systematic review with meta-analysis was to determine a causal relationship between psychological stress and symptom exacerbation in IBD, subsequently utilising Bradford Hill’s criteria (approach never used in this topic area before) to evaluate the likelihood of causal associations.

**Methods:**

Medline, EMBASE, CINAHL and PsycInfo were searched for relevant studies up to July 20, 2019. Data extraction and quality appraisal were performed by two independent reviewers. Results of all retained papers were presented as a narrative synthesis. A random-effect meta-analysis was conducted on studies meeting the criteria for meta-analysis. Bradford Hill criteria were applied to assess the causality of the relationship between all psychological factors and symptom exacerbation.

**Results:**

The searches yielded 2472 potential articles. Nineteen clinical prospective cohort studies were eligible for the narrative review with five suitable for the meta-analysis. Meta-analysis showed depression, anxiety and perceived stress did not have a statistically significant association with an increased risk of symptom exacerbation. Four of the Bradford Hill criteria were met which indicates that there is weak to moderate evidence of a causal association between all the psychological factors and disease activity. Inconsistent results and a dearth of studies using the same tools for measuring psychological factors suggest the need for more research to be done to facilitate more conclusive findings.

**Conclusions:**

This original review utilising Bradford Hill criteria in addition to meta-analysis to evaluate the causality of relationship between psychological factors and symptom exacerbation in IBD provides evidence that psychological factors have a weak to moderate causal involvement in IBD symptom exacerbation. However, when combining this finding with the outcomes of the meta-analysis, we can say that the results were inconclusive**.** Interventions to reduce the associated psychological impact should be part of the treatment plan for patients with IBD.

**Systematic review registration:**

PROSPERO CRD42012003143

## Background

Inflammatory bowel disease (IBD) is a relapsing, chronic condition with unidentified aetiology affecting predominantly the gastrointestinal tract [1]. The condition affects around 2.2 million people across Europe and 28 million people worldwide with an increasing prevalence [2, 3]. Main symptoms include abdominal pain, bloody diarrhoea and nutritional failure, but patients can also suffer from ocular, musculoskeletal and skin pathologies [4]. There is no cure for IBD and a high proportion of patients need hospitalisation and require surgery at 10 years [5]. A combination of highly distressing symptoms, possible hospitalisation and surgery, as well as no imminent prospects of cure impacts on patients’ quality of life.

Despite improvements in pharmacological interventions and surgical outcomes, patients with IBD report a high degree of psychological symptoms associated with the disease [6]. Together, the symptoms impact on all aspects of their lives, often affecting their relationships and employment [7]. In addition, the rising prevalence and incurability of the condition contributes to rising health care costs and has an effect on the health care system [8].

Whilst it has been noted that a high portion of patients with IBD experience psychological comorbidities [9], it is not clear how psychological factors are related to IBD and its disease course. Clinicians and patients have long suspected that there is a relationship between psychological factors and symptom exacerbation in IBD [10, 11]. This is supported by studies in other long-term conditions [12–14]. From evidence we know that psychological stress responses are known to stimulate the production of inflammatory markers in a number of long-term conditions [15, 16]. However, the research investigating psychological factors and its relationship with disease symptoms in IBD has been somewhat conflicting. Some have found an association between psychological factors and exacerbation of IBD symptoms [17–19], whilst others have not [20–22]. As discussed previously in the protocol for this review [23], these contradictory findings could be the result of methodological limitations. For example, some studies have used retrospective data (influenced by recall bias) rather than gathering prospective data [24]. Others have conducted systematic reviews in an attempt to clarify the conflicting findings [25–27]. However, there are issues with methodological quality in previous reviews. For example, some reviews have not been systematically conducted or reported and therefore increase the risk of bias and/or the potential for replicability [25–28]. Other researchers have also used tools with no validity or reliability [29] or have only reported on depression without considering other psychological factors [30]. Thus, these limitations might provide explanation for the ambiguous findings around a causal relationship between psychological factors and symptom exacerbation in IBD.

The challenge of establishing a causal relationship between two variables is not new. In a classic study, Bradford Hill [31] proposed a set of criteria, namely the Bradford Hill criteria, to evaluate systematically whether there is a causal link between an exposure of interest and a health outcome. These criteria have been used by epidemiologists to test causal hypotheses. The named criteria which have stayed virtually unchanged since its first publication are as follows: strength of the association, consistency of findings, specificity of the association, temporal sequence association, biological gradient, biological plausibility, coherence and experiment. Applying these criteria will help to reduce ambiguity around the relationship between psychological factors and symptom exacerbation of IBD.

Establishing a causal link between psychological factors and symptom exacerbation of IBD would ensure that the correct treatment interventions are available, which could potentially reduce perpetual flare-ups and associated distressing symptoms. Thus, the overall aim of this systematic review is to examine if there is a causal link between psychological factors and symptom exacerbation in IBD by utilising the Bradford Hill criteria, which have never been used in this topic area to date.

## Methods

This systematic review and meta-analysis were undertaken according to the Preferred Reporting Items for Systematic Reviews and Meta-Analyses (PRISMA) statement [32].

### Search strategy

Systematic searches of published papers indexed in MEDLINE (via Ovid), MEDLINE (via PubMed), EMBASE (via Ovid), CINAHL (via Ebsco) and PsycInfo (via Ebsco) were searched for relevant articles published in English from commencement of databases to July 20, 2019. In addition, hand searches of the reference list were conducted of the relevant articles to identify any other relevant studies missed by the previous searches.

The search strategy was designed with input from a health specialist subject librarian. The following search terms and their MeSH (medical subject heading) equivalents were used: inflammatory bowel disease, Crohn’s disease, ulcerative colitis, psychological stress, mental stress, life stress, family stress, hassles, social stress, psychological distress, perceived stress, mood disorders, anxiety, depression and personality. The search strategy developed for Medline (see list below) was amended and used in the other databases. The sample of search strategies for EMBASE, Cinahl and PsycInfo are in supplementary files.

### Search strategy for MEDLINE

1.Inflammatory Bowel Diseases/ or Inflammatory Bowel.mp.

2.Crohn’s Disease/

3.Colitis, Ulcerative/

4.Stress, Psychological/

5.mental stress.mp.

6.life stress.mp.

7.family stress.mp.

8.hassles.mp.

9.social stress.mp.

10.coping.mp.

11.perceived stress.mp.

12.mood disorders.mp. or Mood Disorders/

13.Anxiety/

14.Depression/

15.4 or 5 or 6 or 7 or 8 or 9 or 10 or 11 or 12 or 13 or 14

16.1 or 2 or 3

17.15 and 16

### Study selection

Studies were included in this review if they met the following criteria: (1) prospective cohort studies that reported on a causal association between psychological factors and symptom relapse in IBD patients; (2) participants of age 18 or above and with a diagnosis of Crohn’s disease or ulcerative colitis; (3) reporting on psychological factors where they are clearly defined and the measurement tools used were clearly identified; (4) reporting on symptom exacerbation (flair, symptom relapse or disease activity) and explicitly giving details on tools used to measure disease activity/symptom relapse which is the opposite of symptoms remission and (5) published in English. The rationale for the inclusion criteria was published in the study protocol [23]. In the instances where there was overlap of data within and between the studies, only those reporting the longest duration of follow-up or the largest number of participants were included. A broad definition of psychological stress was adopted, to include all the variety of minor to major psychological factors (psychological stress, mental stress, life stress, family stress, hassles, social stress, psychological distress, perceived stress, mood disorders, anxiety, depression, personality).

The study selection was in two stages. Firstly, two reviewers (MS and MB) independently screened all titles and abstracts applying the pre-set screening checklist presented in Table 1. Then the two reviewers independently screened the full text of the potentially relevant papers. Any disagreements were resolved by discussion with a third reviewer (TG). 
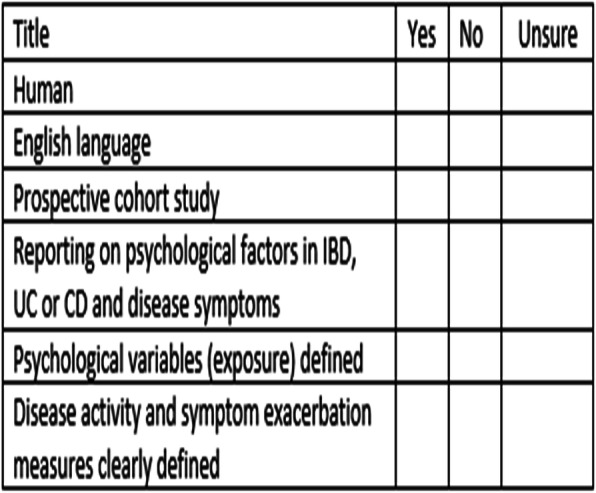

**Table 1:** Screening checklist

Title	Yes	No	Unsure
Human			
English language			
Prospective cohort study			
Reporting on psychological factors in IBD, UC or CD and disease symptoms			
Psychological variables (exposure) defined			
Disease activity and symptom exacerbation measures clearly defined			

### Data extraction and quality appraisal

Data from each paper was extracted independently by 2 reviewers (from MS, MB, TG) using a review specific extraction tool. The extracted data included details on the first author, year published, country, study and methods, study populations with disease type, follow-up, types of stress exposure, quality rating and statistical assessment (Table 3). All extracted data was cross-checked and disagreements were resolved by consensus among the researchers. In cases of missing data, authors were contacted and asked to provide the missing information. The methodological quality of all papers meeting the eligibility criteria was assessed independently by two researchers (MS and MB) using the Critical Appraisal Skills Programme (CASP) tool for cohort studies [33] (supplementary files). This tool is widely used for critically appraising cohort studies and consists of 14 questions of which most can be answered with ‘yes’, ‘no’ or ‘don’t know’. The two researchers assessed the quality of all papers before determining whether papers presented a high or low risk of bias. Studies with a low risk of bias were included in the review.

### Data presentation and synthesis

As per Centre for Reviews and Dissemination-CRD [34], a summary of extracted data from included studies is presented in tabular form as part of the review (Table 3). We provided synthesis utilising the Bradford Hill criteria of causation for each psychological factor.

### Analysis

The following Bradford Hill criteria for causation between psychological factors and symptom exacerbation in IBD were evaluated for each of the psychological stress categories:

1. *Strength and association*
2. *Temporality*
3. *Coherence*
4. *Consistency*
5. *Plausibility*
6. *Biological gradient*
7. *Experiment*

Only four criteria were deemed applicable. Specificity was not evaluated because single exposure to psychological factors and outcome of symptom relapse does not preclude a causal relationship. The four Bradford Hill criteria were used to calculate a causation score for each psychological factor.

1. The principles used to evaluate/compute the criteria *strength of associations*, used previously by Roffey et al. [35] are summarised in Table 2 (below). A score of 1 was given for moderate to strong strength of association and a score of 0 was given for none to a weak association (Table 4). Two reviewers assessed all studies and agreed on application of the criteria on all the studies.
2. *Temporality* or temporal sequence of association means that exposure (psychological stress) must precede outcome (symptom exacerbation).
3. *Coherence* refers to whether similar conclusions have been drawn across all the studies in the review.
4. *Consistency.* This criterion examines if the same findings have been observed among different populations, in different study designs and different times.
5. *Plausibility* is looking at the presence of a potential biological mechanism of causality.
6. *Biological gradient* examines if the changes in disease (symptom) activity corresponds to changes in exposure (length or intensity of exposure to psychological factors).
7. *Experiment* examines if the removal of the exposures (psychological factors) will alter the frequency of the outcome. 
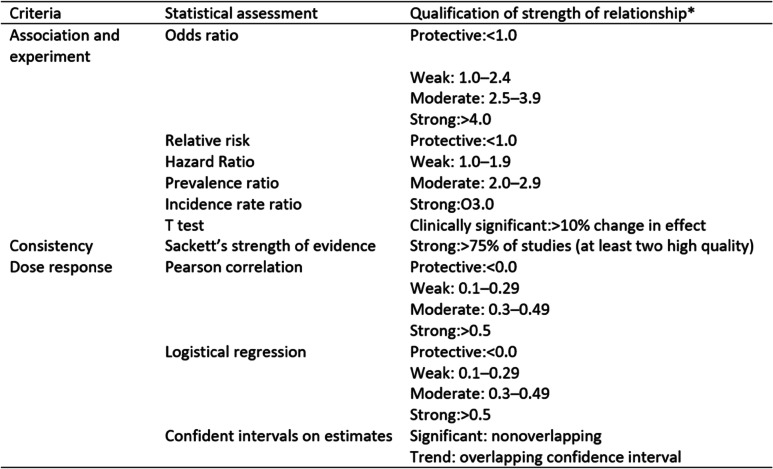

**Table 2:** Statistical assessment for specific Brad Hill criteria for causation

Criteria	Statistical assessment	Qualification of strength of relationship^a^
Association and experiment	Odds ratio	Protective:<1.0
		Weak: 1.0–2.4
		Moderate: 2.5–3.9
		Strong:>4.0
	Relative risk	Protective:<1.0
	Hazard Ratio	Weak: 1.0–1.9
	Prevalence ratio	Moderate: 2.0–2.9
	Incidence rate ratio	Strong:O3.0
	T test	Clinically significant:>10% change in effect
Consistency	Sackett’s strength of evidence	Strong:>75% of studies (at least two high quality)
Dose response	Pearson correlation	Protective:<0.0
		Weak: 0.1–0.29
		Moderate: 0.3–0.49
		Strong:>0.5
	Logistical regression	Protective:<0.0
		Weak: 0.1–0.29
		Moderate: 0.3–0.49
		Strong:>0.5
	Confident intervals on estimates	Significant: nonoverlapping
		Trend: overlapping confidence interval

^a^Strength at the risk estimate level refers to how strong a relationship is for the observed unique risk estimate or comparison

Each of the Bradford Hill criteria was allocated a value of 1 if the criteria were satisfied and a value of 0 if the criteria were not satisfied (Table 4). Points were then added to give an overall causation score (range 0–7) for each association as per Degelman [36]. Scores of 6 or 7 represent strong causal association, whilst scores of 4 or 5, and ≤ 3 represent moderate and weak causal association respectively. It is important to point out that the causation score is different to the strength of association score (Table 2) as the latter refers to the strength of the relationship for the observed unique risk estimate or comparison. A similar evaluation process using different scoring systems has been employed previously by others [36–38].

### Meta-analysis and heterogeneity

We performed random-effect meta-analysis on the qualifying studies in order to pool the estimates of association. We computed the direction and effect size of the impact of perceived stress, depression and anxiety on disease activity using the MetaXL Version 5.3 software for meta-analysis [39]. Hazard ratios (HRs) were used as common risk estimates across the studies. Forest plots were produced to visually access the association across the studies and the corresponding 95% CI. I-squared statistics and chi-squared test were used to assess heterogeneity. Random-effect models are preferred over fixed-effects models when we have heterogeneity. We initially conducted both types to assess the robustness of our estimates, which revealed consistent conclusions. The results from random-effect models were presented for consistency.

## Results

### Literature search

**Fig. 1:**
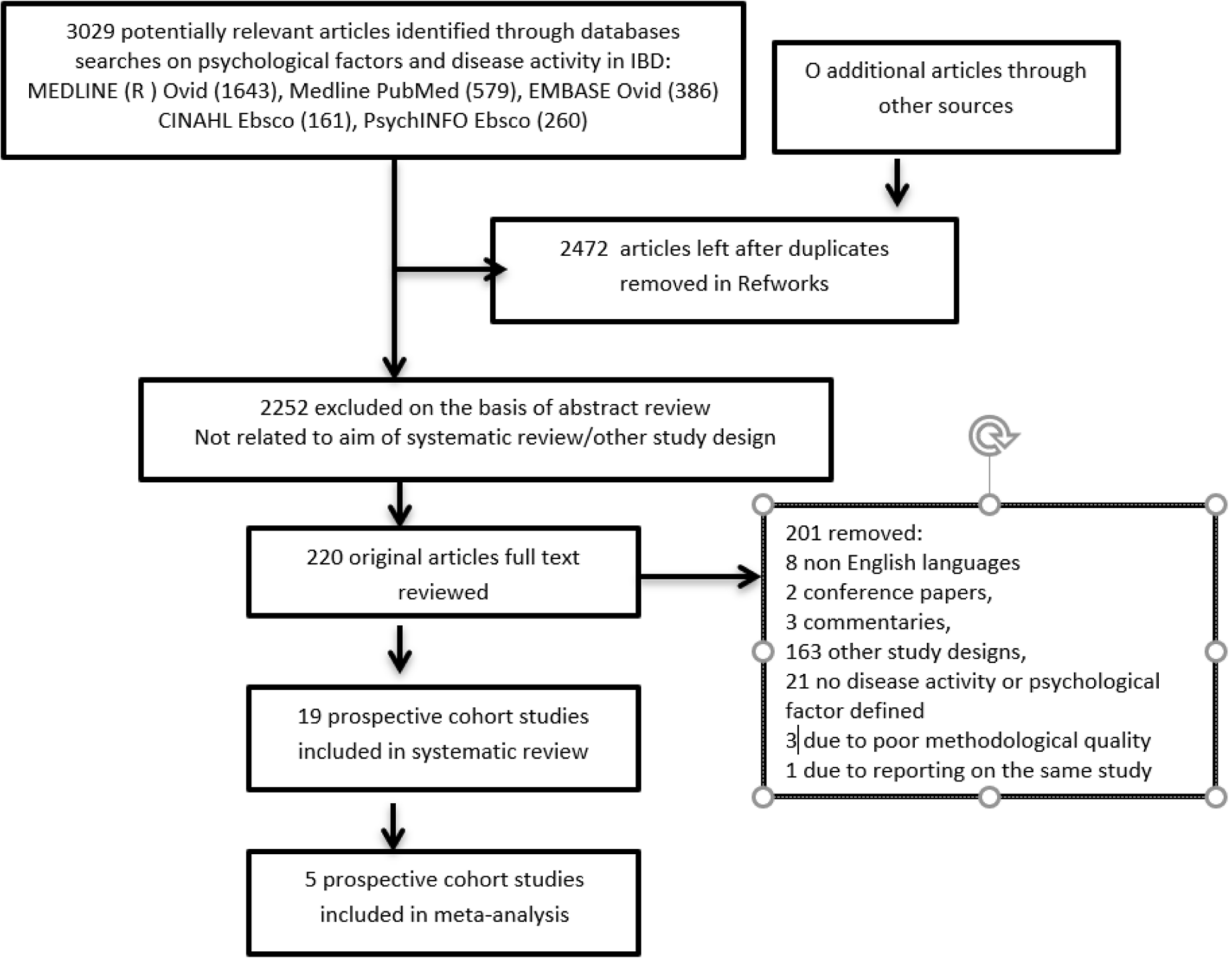
PRISMA flow diagram of studies

The search strategy identified 2472 potential studies, of which 19 were included in the systematic review (10,188 participants in total). All database results were imported in RefWorks where duplicates were removed. See Fig. 1 for further details.

### Application of CASP

The results of the application of CASP found that most papers were of high quality (see Additional File for CASP Results). The responses of the questions were used to determine whether decisions made or items missing would potentially have a high bias affecting the findings. For example, we considered whether the generalisability of findings could be affected by the recruitment of the cohort, the validity and reliability of the instruments used to measure disease and psychological factors and how cofounding variables were managed. Overall, the analyses highlighted the need for improved reporting. In particular, there was limited detail of statistical analysis. Three studies [24, 29, 40] were removed from the review due to their high risk of bias. For example, participants collected data for the first week of every month and this was presented as data for that particular month. This does not take account of the fluctuating disease symptoms of IBD. Following application of CASP 19 papers were retained.

### Study characteristics

Characteristics of the included studies are presented in Table 3. Ten papers originated from North America, seven from Europe and one each from South America and Australia. The cohort size ranged from *n* = 18 for smallest cohort [41] to *n* = 4314 for the largest cohort [42]. Of the 19 studies, 11 were looking at the combined IBD population, 5 focused on CD patients only and 3 looked at UC patients. All studies included adults aged 18 and over. The shortest cohort follow-up was 3 month [43] and the longest one was 108 months [44]. Characteristics of studies psychological factors are shown in Table 3. Out of all the 19 studies, the most commonly measured psychological factors were as follows: depression (14), perceived stress (9) and anxiety (9). The rest of the stressors were as follows: major life stress, recent life events, daily strains, low mood and high mood, hopelessness, psychological distress and long-term stressors. All studies measured psychological factors through self-reported questionnaires. Disease activity/symptom relapse was assessed in a number of different ways (see Table 3 for details). All included studies were of high methodological quality.

**Table 3:** Characteristics of included cohort studies

First author and year published	Country	Cohort size (*n*)	Disease	Follow-up in months	Stress exposure	Disease activity	Stats assessment	Strength of association
Bernstein 2010	Canada	552	IBD	12	Major life stress NRS Perceived stress CPSS Low positive mood PANAS High negative mood PANAS	Manitoba IBD index	OR = 1.69 (1.13,2.54) OR = 2.63 (1.72,4.01) OR = 1.42 (0.94,2.15) OR = 1.73 (1.13,2.66)	Weak Moderate Weak Weak
Duffy 1992	USA	124	IBD	11.5	Major life stress SRE Daily strains Checklist Perceived Stress ladder	mCDAI	*R* = .32, *p* < .001* *R* = .20, *p* < .05 *R* = .31, *p* .001	Moderate Weak Moderate
Mardini 2004	USA	18	CD	24	Depression BDI Anxiety BAI Hopelessness BHS Recent Life Change RlCQ	CDAI	*β* = 5.92 *p* = .0004 *β* = 2.42 *p* = .02 *β* = 4.87 *p* = .05 *β* = 0.08 *p* = .10	Strong Strong Strong Weak
North 1991	USA	32	IBD	24	Depression CD mBDI Depression UC mBDI Recent life events CD SRRS Recent life events UC SRRS	GSS	*β* = − 0.03–0.56 (− 0.57–1.05)** *β* = − 0.53–1.34 (− 1.84–2.63) *β* = 0.17–0.34 (0.32–.75) *β* = − 1.47–1.66(− 2.61–4.69)	Strong Strong*** Moderate Strong***
Mikocka-Walus 2008	Australia	127	IBD	12	Depression HADS, SCL Anxiety HADS, SCL	CDAI, SCCAI	OR = 1.003 (0.928–1.085) OR = 1.057(0.919–1.215) OR = 1.040 (0.989–1.092) OR = 0.967(0.841–1.111)	Weak Weak Weak Protective
Mittermaier 2004	Austria	60	IBD	18	Perceived stress V PSQ Perceived stress G PSQ Depression BDI Anxiety STAI	CDAI, CAI	TAUR = 0.1146 *R* = .0226 *R* = .2023 *p* = < .05 *R* = .1844–.1981 *p* = < .05	Weak None Weak Weak
Vidal 2006	Spain	155	IBD	11	Recent life events SRRS	IBD activity index CDAI/TLWI, HBi	HR = 0.88 (0.68–1.13)	None
Vidal De Lima 2012	Brazil	50	CD	16	Depression and anxiety BDI HADS	CDAI	*p* = .15	None
Bitton 2008	Canada	87	CD	12	Daily hassle DHS Perceived stress PSS Psychological distress Depression SCL Anxiety SCL	CDAI	HR = 1.05 (0.95 to 1.15) HR = 1.2 (0.9 to 1.6) HR = 1.0(0.85 to 1.2) HR = 1.6 (0.9 to 2.7) HR = 1.4 (0.77 to 2.6)	Weak Weak Weak Weak Weak
Bitton 2003	USA	60	UC	12	Psychological distress Depression SCL Anxiety SCL Perceived stress PSS Recent stress events PERI	Endoscopygrading scale	HR = 1.038(0.95–1.39) HR = 1.011 (0.95–1.08) HR = 1.000 (1.00–1.00) HR = 0.898 (0.53–1.53) HR = 1.165 (0.98–1.39)	Weak Weak Weak Weak None
Duffy 1991	USA	124	IBD	6	Major stress events SRE Health-related stress	CDAI	RR = 2.6 (1.3–4.9) RR = 3.8 (1.5–9.9)	Moderate Strong
Langhorst 2013	Germany	75	UC	12	Perceived stress PSS Depression and anxiety SCL	CAI	HR = 0.20(0.01–3.31) HR = 1.05 (1.01–1.10) HR = 1.08 (0.95–1.22)	None Weak Weak
Levenstein 2000	Italy	62	UC	45	Major life stress PLEI Perceived stress LT PSQ Depression CES-D	CRP/Rectal biopsy	HR = .73 (0.27–2.0) HR = 2.8 (1.1–7.2) HR = 0.99 (0.36–2.7)	None Strong None
Camara 2011	Switzerland	468	CD	18	Perceived stress G-PSQ Anxiety model HADS Depression model HADS	CDAI	OR = 1.85 (1.43–2.40) OR = 1.78 (1.38–2.30) OR = 1.78 (1.38–2.28)	Weak Weak Weak
Bernstein 2016	Canada	487	IBD	3	Perceived stress PSS	MIBDI	*R* = .71–.78 (.52–.88)	Strong
Gaines, 2016	USA	2144	CD	12	Depression PROMIS	SCDAI	*t* (*p* = 0.001, df = 2)	Weak
Mikocka-Walus 2016	Switzerland	2007	IBD	108	Depression IBD HADS Depression CD HADS Depression UC HADS Anxiety IBD HADS Anxiety CD HADS Anxiety UC HADS	Physician Assessed CDAI MTWAI	*p* = .000001 *p* = .0007 *p* = .005 *p* = .0014 *p* = .031 *p* = .066	Strong Strong Strong Strong Strong Weak
Gracie 2018	UK	405	IBD	24 months	Depression HADS Anxiety HADS	HBI SCCAI	HR = 0.86; (0.33–2.27) HR = 2.08 (1.31–3.30)	Weak Moderate
Kochar 2018	USA	2798 1516	CD UC	22 months 24 months	Depression PHQ Depression PHQ	mHBI SCCAI	RR = 2.3 (1.9–2.8) RR = 1.3 (0.0–1.7)	Moderate Weak

*NRS* numerical rating scale, *CPSS* Cohen Perceived Stress Scale, *PANAS* Positive and Negative Affect Schedule, *SRE* Schedule of Recent Experiences, *mCDAI* modified Crohn’s disease activity index, *BDI* Becks Depression Inventory, *BAI* Becks Anxiety Inventory, *BHS* Becks Hopelessness Scale, *RLCQ* Recent Life Change Questionnaire, *CDAI* Crohn’s disease activity index, *mBDI* modified Becks Depression Inventory, *SRRS* stressful life events and hassles, *TLWI* Truelove-Witts index, *GSS* gastrointestinal symptom scale, *HADS* Hospital Depression and Anxiety Scale, *SCL* Symptom Checklist Scale, *SCCAI* Simple Clinical Colitis Activity Index, *PSQ* Perceived Stress Questionnaire, *CES-D* Center for Epidemiological Studies Depression Scale, *CRP* clinical rigid proctoscopy, *CAI* Colitis Activity Index, *HBI* Harvey-Bradshaw Index, *DHS* Daily Hassle scale, *PSS* Perceived Stress Scale, *PERI* Psychiatric Epidemiology Research Interview, *PLEI* Paykel Life Experiences Interview, *MIBDI* Manitoba Inflammatory Bowel Disease Index, *PROMIS* Patient reported Outcomes Measurement Information System, *MTWAI* Modified Truelove and Witts Severity Index, *PHQ* Patient Health Questionnaire, *mHBI* modified Harvey-Bradshaw index, *SCAI* Simple Colitis Activity Index. *Β* regression coefficient, *R* correlation coefficient

*Correlation coefficient *R* + 0.30. A weak uphill (positive) linear relationship. + 0.50. A moderate uphill (positive) relationship

**p = .028

***Some negative associations

### Depression on symptom exacerbation in IBD

Fourteen out of the nineteen studies looked at depression among IBD patients. Five studies [22, 43, 45–47] used the Hospital Anxiety and Depression Scale (HADS) and three used Becks Depression Inventory (BDI) or a modified version of it [41, 48, 49]. The rest of the studies used the Centre for Epidemiological Studies Depression Scale (CES-D) [50]; Symptom Checklist-90R SCL [1, 18, 22], Patient Reported Outcomes Measurement Information Systems (PROMIS) [51] or the Patient Health Questionnaire (PHQ-8) [42]. Five studies [1, 41, 46, 51, 52] looked at CD patients, three studies looked at UC [18, 45, 50], whilst the rest looked at mixed sample of IBD patients.

### Perceived stress on symptom exacerbation in IBD

Nine of the nineteen studies looked at perceived stress among IBD patients. Five studies used the Cohen Perceived Stress Scale (CPSS) to measure perceived stress [1, 18, 19, 43, 45]. Three studies used the Perceived Stress Questionnaire (PSQ) [46, 49, 50]. One study used the stress ladder to assess perceived stress [53]. Two studies [1, 46] looked at CD patients, three studies looked at UC [18, 45, 50] and four studies looked at a mixed sample of IBD patients [19, 43, 49, 53]. Most of the studies found that perceived stress was associated with subsequent symptomatic flare [1, 18, 19, 43, 45, 46, 50, 53]. Out of these, Langhorst et al. [45] found that short-term stress (including acute perceived stress) to be related to relapse, whilst Levenstein et al. [50] found for long-term stress increases the risk of exacerbation.

### Anxiety on symptom exacerbation in IBD

Nine of the nineteen studies looked at anxiety [1, 18, 22, 41, 44, 46, 47, 49, 52]. Four studies measured anxiety with HADS [22, 44, 46, 52] and three studies used the STAI, BAI and PHQ-8 [41, 47, 49]. Five studies looked at CD [1, 41, 42, 46, 52], two focused on UC [18, 42] and four studies [22, 44, 47, 49] looked at IBD.

### Major life stress on symptom exacerbation in IBD

Four of the nineteen studies looked into the association between major life events and IBD. Two studies [53, 54] used the Schedule of Recent Experiences to assess major life stress, one study used a numerical rating scale to describe the stress impact with 0 = not at all stressful to 10 = extremely stressful [19], and one study [50] used the Paykel Life Experiences Interview. One study was in UC [50] and three were in IBD [19, 53, 54].

### Recent life events on symptom exacerbation in IBD

Four studies examined the relationship between life events and symptom exacerbation in IBD. Two studies used the Social Readjustment Rating Scale [21, 48], whilst Mardini et al. [41] used Holmes Recent Life Changes (RLC) and Bitton et al. [18] used the Psychiatric Epidemiology Research Interview (PERI) Life events scale. All the questionnaires used were designed to measure the degree of psychological distress, as opposed to being used for diagnostics assessment. Two studies included both CD and UC patients [21, 48], one studied only UC [18] and one only CD [41].

### Daily strains on symptom exacerbation in IBD

Two studies looked at daily strains on symptom exacerbation in IBD [53] and CD [1]. Duffy at al [53]. refers to daily strains as to the day-to-day hassles and major events persisting for longer than 3 months. Duffy et al. [53] measured these with an unnamed checklist, previously described in Kanner et al. [55] and Thoits et al. [56]. Bitton et al. [1] assessed minor life stress using a version of the Hassles Scale, asking participants to rate each of the 53 minor events in the scale during the past month on a 4-point (0–3) scale.

### Low and high affect (mood) on symptom exacerbation in IBD

Only one study looked at low and high affect (mood) on symptom exacerbation in IBD [19]. Positive and negative emotional styles were evaluated using the Positive and Negative Affect Schedule (PANAS). Disease activity was measured by the Manitoba Inflammatory Bowel Disease Index (MIBDI). This study found that those with persistently active disease were more likely to report to have low positive affect (55.6 % vs. 40.3%, *p* = 0.02), and to have high negative affect (67.1% vs. 36.9%, *p* < 0.0001) when compared to those without active disease.

### Hopelessness on symptom exacerbation in IBD

Mardini et al. [41] was the only study to look at the relationship of hopelessness and disease activity in CD. They used the Beck Hopelessness Scale (BHS), which is a 20-item questionnaire. The questionnaire can score a maximum of 20 points summarising the responses of hopelessness for each item, consisting of true-or-false statements. Although in this study it was found that increased hopelessness is associated with increased Crohn’s disease activity, the effects were generally of reduced magnitude comparing to the other psychological measures.

### Psychological distress on symptom exacerbation in IBD

Bitton at al [18]. was the only study to examine the relationship between psychological distress and symptom exacerbation in IBD. Specifically, patients with UC completed the Symptom Checklist-90R, a 90 item self-report measure that assesses symptoms of distress and Global Severity Index. No significant association was found between symptom relapse and PSS scores.

### Long-term stressors on symptom exacerbation in IBD

Langhorst et al. [45] was the only study to include a measure of long-term stressors and symptom exacerbation. Patients with UC were followed up for 12 months and completed the Perceived Stress Questionnaire (PSQ) at baseline, then 3, 6, 9 and 12 months respectively. The PSQ captures a subjective interpretation of the frequency of historical stressful events. Patients rated how often an item applied to them on a 4-point scale (1 = almost never and 4 = usually). No validation studies had previously determined a cutoff point for elevated long-term stress; therefore, the researchers of this study defined elevated long-term stress by score > 1 SD than the mean value of a health population.

### Results from the pooled analysis

**Fig. 2:**
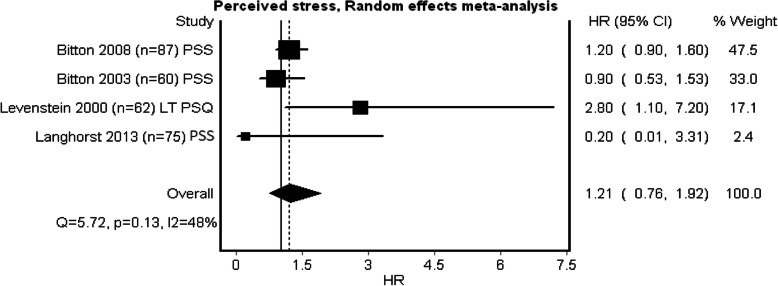
Forest plot for the effect of perceived stress on symptom exacerbation in IBD. Tools to measure perceived stress: *PSS* Perceived Stress Scale, *PSQ* Perceived Stress Questionnaire

**Fig. 3:**
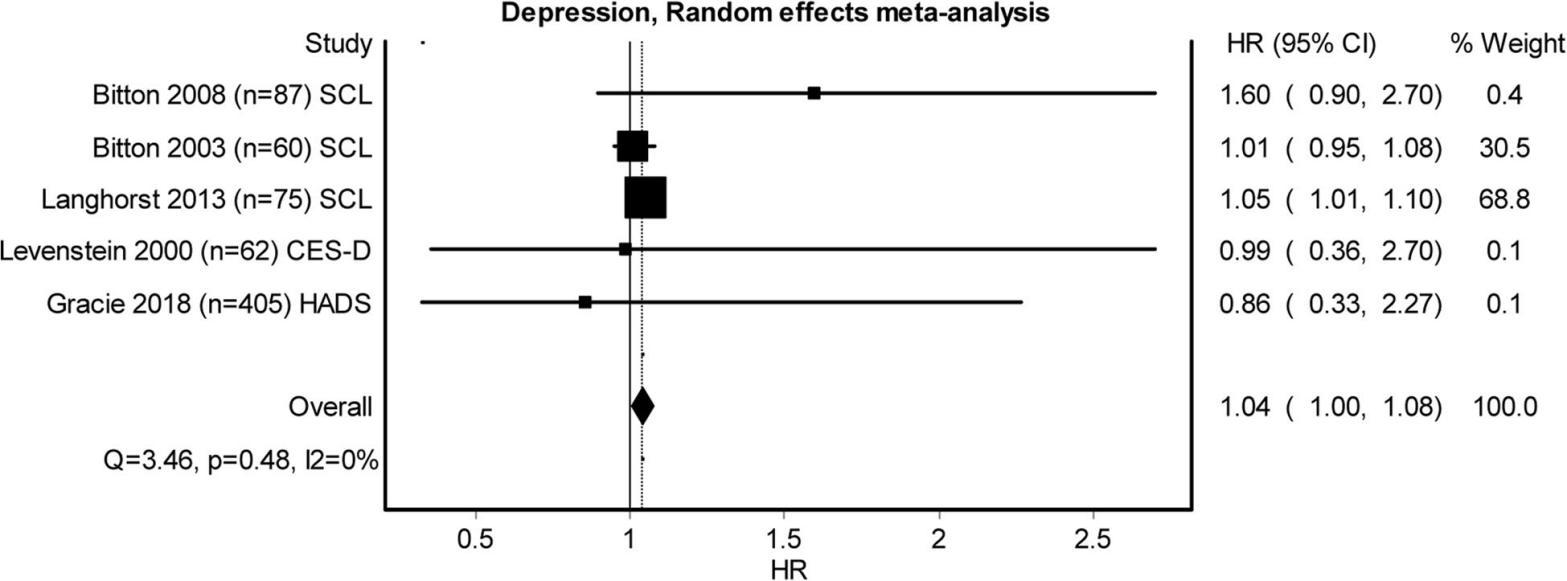
Forest plot for the effect of depression on symptom exacerbation in IBD. Tools to measure depression: *SCL* Symptom Checklist Scale, *CES-D* Center for Epidemiological Studies Depression Scale, *HADS* Hospital Anxiety and Depression Scale

**Fig. 4:**
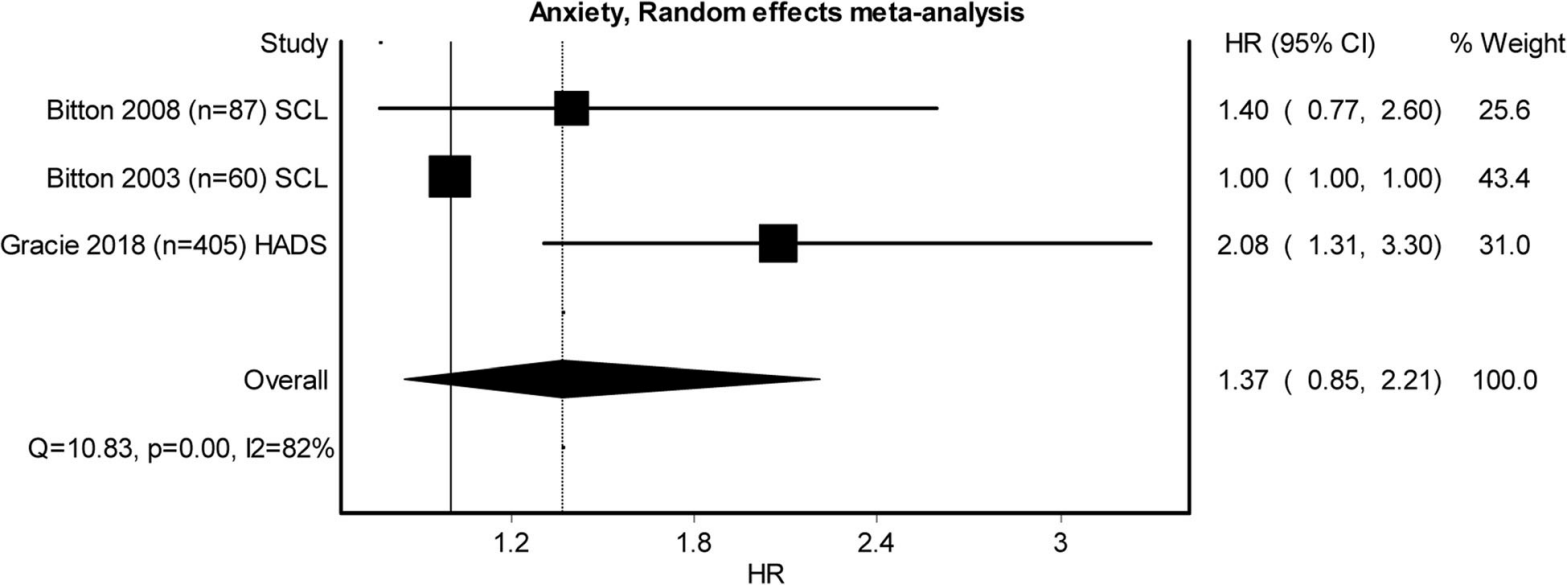
Forest plot for the effect of anxiety on symptom exacerbation in IBD. Tools to measure anxiety: *SCL* Symptom Checklist Scale, *HADS* Hospital Anxiety and Depression Scale

**Fig. 5:**
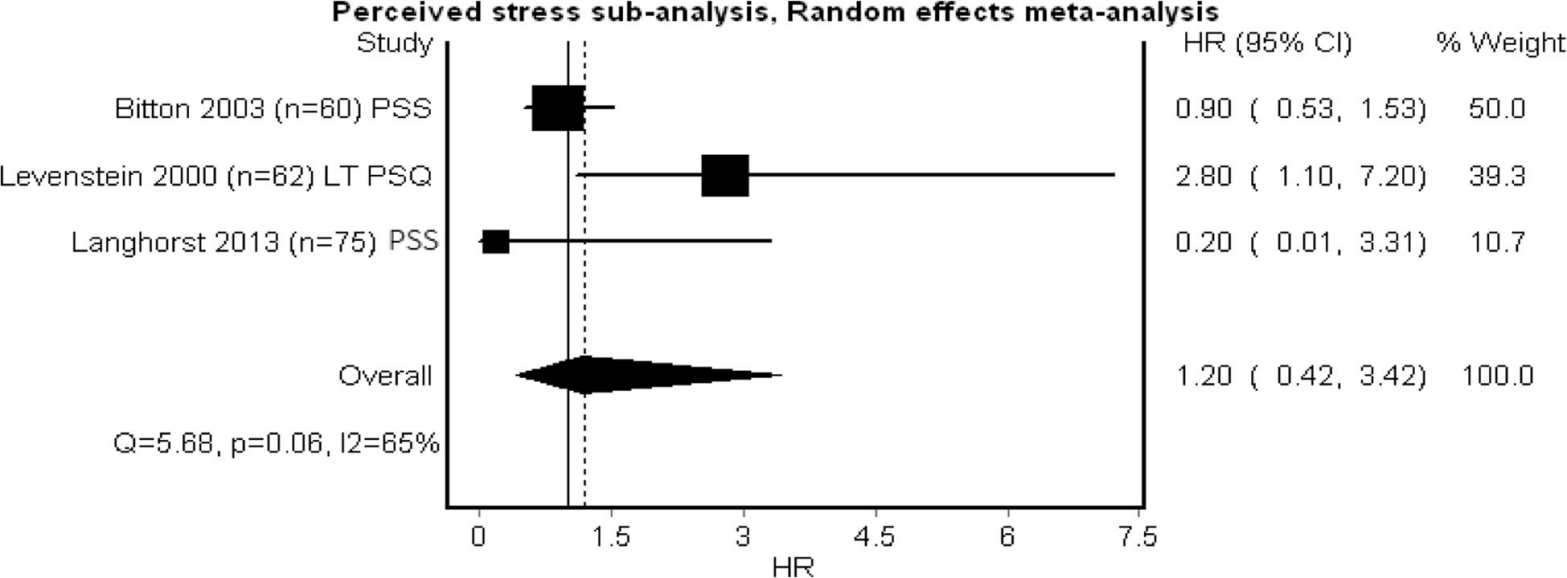
Forest plot of perceived stress on symptom exacerbation in UC (subgroup analysis). Tools to measure perceived stress: *PSS* Perceived Stress Scale, *PSQ* Perceived Stress Questionnaire

**Fig. 6:**
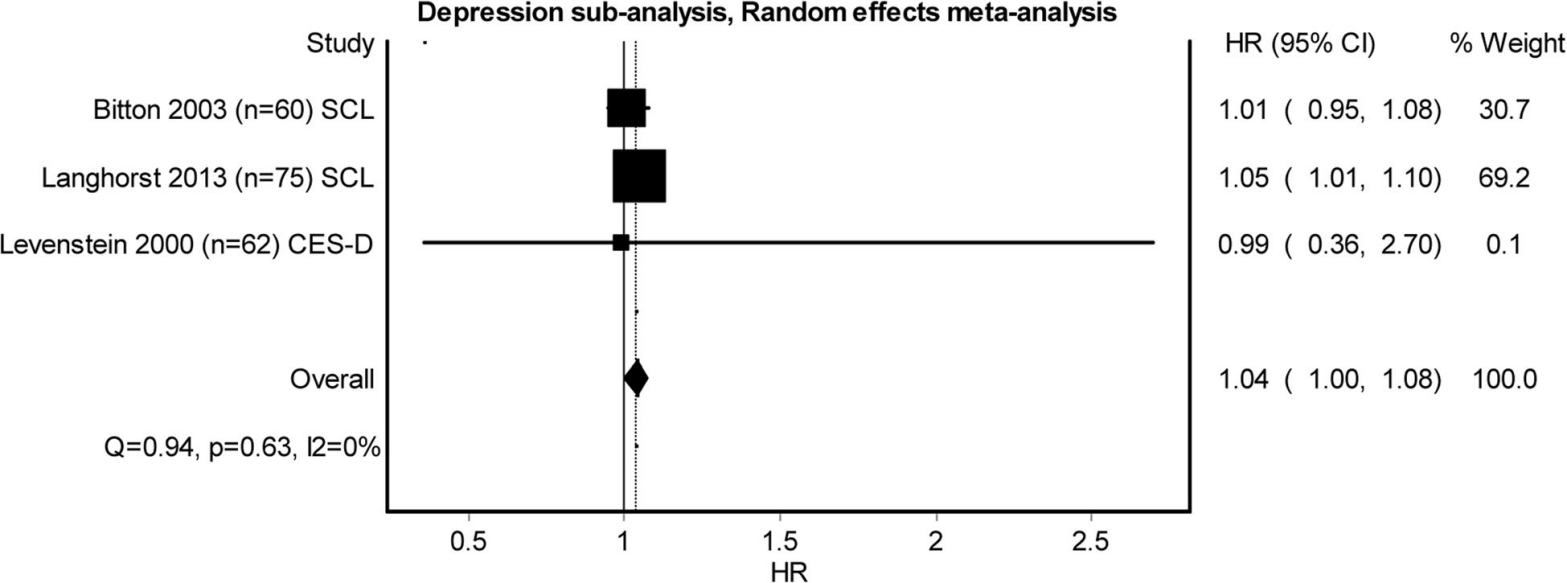
Forest plot for the effect of depression on symptom exacerbation in UC (subgroup analysis). Tools to measure depression: *SCL* Symptom Checklist Scale, *CES-D* Center for Epidemiological Studies Depression Scale

Three studies examining perceived stress, five examining depression and three examining anxiety qualified to be entered in the pooled meta-analysis (see Figs. 2, 3 and 4). Heterogeneity was minimal in perceived stress (*Q* = 4.27, *p* = .12, *I*^2^ = 53%) and depression (*Q* = 3.46, *p* = .48, *I*^2^ = 0%), whilst heterogeneity for anxiety was substantial (*Q* = 10.84, *p* = 0.00, *I*^2^ = 82%). Whilst all the pooled analysis of combined HR for the studies showed impact of baseline psychological stress on symptom exacerbation, none of them showed a significant statistical effect over symptom exacerbation (Figs. 2, 3 and 4). Subgroup analyses looking at separate disease types were conducted. The sub-analysis was only possible for perceived stress and depression in UC as there was more than one study. Sub-analysis was not performed for anxiety, as there was only one study. The subgroup analysis for perceived stress and depression in UC only (Figs. 5 and 6) did not show any significant statistical effect of perceived stress or depression over symptom exacerbation (pooled perceived stress HR 1.20, 95% CI 0.42–3.42 and pooled depression HR 1.04, 95% CI 1–1.08).

### Consideration against the Bradford Hill criteria for determining causality

The analysis for each of the Bradford Hill aspects of causation is outlined in Table 4 which provides details about the strength of association for each of the psychological factors examined in the 19 studies as well as the value for the other Hill criteria.

**Table 4:** Assessment of observed data from cohort studies with Bradford Hill criteria for assessing a potential causal relationship between psychological stress and symptom exacerbation

Exposure	Studies	Disease	Cohort size	Strength of association	Temporality	Coherence	Consistency	Biological gradient	Experiment	Plausibility	Criteria met
Depression Depression Depression Depression Depression Depression Depression Depression Depression Depression Depression Depression Depression Depression Depression	Mardini 2004 North 1991 Mikocka-Walus 2008 Mittermaier 2004 Vidal De Lima 2012 Bitton 2007 Bitton 2003 Langhorst 2013 Levenstein 2000 Camara 2011 Gaines 2016 Mikocka-Walus 2016 Gracie 2018 Kochar 2018 Kochar 2018	CD IBD IBD IBD CD CD UC UC UC CD CD IBD IBD CD UC	18 32 127 60 50 87 60 75 62 468 2144 2007 405 2798 1516	Strong = 1 Strong = 1 Weak = 0 Weak = 0 None = 0 Weak = 0 Weak = 0 Weak = 0 None = 0 Weak = 0 Strong = 1 Strong = 1 Weak = 0 Moderate = 1 Weak = 0	1 1 1 1 1 1 1 1 1 1 1 1 1 1 1	N/A	1 1 1 1 1 1 1 1 1 1 1 1 1 1 1	N/A	N/A	1 1 1 1 1 1 1 1 1 1 1 1 1 1 1	4 4 3 3 3 3 3 3 3 3 4 4 3 4 3
Perceived stress Perceived stress Perceived stress Perceived stress Perceived stress Perceived stress Perceived stress Perceived stress Perceived stress	Bernstein 2010 Duffy 1992 Mittermaier 2004 Bitton 2007 Bitton 2003 Langhorst 2013 Levenstein 2000 Camara 2011 Bernstein 2016	IBD IBD IBD CD UC UC UC CD IBD	552 123 60 87 60 75 62 468 487	Moderate = 1 Moderate = 1 Weak = 0 Weak = 0 Weak = 0 None = 0 Strong = 1 Weak = 0 Strong = 1	1 1 1 1 1 1 1 1 1	N/A	1 1 1 1 1 1 1 1 1	N/A	N/A	1 1 1 1 1 1 1 1 1	4 4 3 3 3 3 4 3 4
Anxiety Anxiety Anxiety Anxiety Anxiety Anxiety Anxiety Anxiety Anxiety	Mardini 2004 Mikocka-Walus 2008 Mittermaier 2004 Vidal De Lima 2012 Bitton 2007 Bitton 2003 Camara 2011 Mikocka-Walus 2016 Gracie 2018	CD IBD IBD CD CD UC CD IBD IBD	18 127 60 50 87 60 468 2007	Strong = 1 Weak = 0 Weak = 0 None = 0 Weak = 0 Weak = 0 Weak = 0 Strong = 1 Moderate = 1	1 1 1 1 1 1 1 1 1	N/A	1 1 1 1 1 1 1 1 1	N/A	N/A	1 1 1 1 1 1 1 1 1	4 3 3 3 3 3 3 4 4
Major life stress Major life stress Major life stress Major life stress	Bernstein 2010 Duffy 1992 Duffy 1991 Levenstein 2000	IBD IBD IBD UC	552 123 124 62	Weak = 0 Moderate = 1 Moderate = 1 None = 0	1 1 1 1	N/A	1 1 1 1	N/A	N/A	1 1 1 1	3 4 4 3
Recent life events Recent life events Recent life events Recent life events	Mardini 2004 North 1991 Vidal 2006 Bitton 2003	CD IBD IBD UC	18 32 155 60	Weak = 0 Moderate = 1 /strong = 1 None = 0 None = 0	1 1 1 1 1	N/A	1 1 1 1 1	N/A	N/A	1 1 1 1 1	3 4 4 3 3
Daily strains Daily strains	Duffy 1992 Bitton 2008	IBD CD	123 87	Weak = 0 Weak = 0	1 1	N/A	1 1	N/A	N/A	1 1	3 3
Low + mood High − mood	Bernstein 2010 Bernstein 2010	IBD IBD	552 552	Weak = 0 None = 0	1 1	N/A	1	N/A	N/A	1	3 3
Hopelessness	Mardini 2004	CD	18	Strong = 1	1	N/A	1	N/A	N/A	1	4
Psychological distress	Bitton 2003	UC	60	Weak = 0	1	N/A	1	N/A	N/A	1	3
Long-term stress	Langhorst 2013	UC	75	None = 0	1	N/A	1	N/A	N/A	1	3

Strength of association: none = 0, weak = 0; moderate = 1, strong = 1. Interpretation of causal association: scores of 6 or 7 = strong, scores of 4 or 5 = moderate and ≤ 3 = weak association

#### Strength of association

All studies provided summary statistics (see Table 3) such as HR, OR or by another test of the statistical significance of association. When the principles used to evaluate/compute the first criteria (strength of associations) as per Roffey et al. [35] (Table 2), the findings were as follows: of the 14 studies reporting on estimates of association for depression, the strength of association was classified as ‘strong’ in 4 (28%) studies, ‘moderate’ in 1 (7%), ‘weak’ in 8 (57%) and ‘none’ in 2 (14%). Of the 9 studies reporting associations of perceived stress and symptom exacerbation, 2 (22%) reported ‘strong’, 2(22%) reported ‘moderate’, 4 (44%) reported ‘weak’ and 1 (11%) reported ‘no’ associations. Of the 8 studies reporting associations for anxiety, 2 (22%) reported ‘strong’, 1 (11%) reported ‘moderate’, 5 (55.5%) reported ‘weak’ and 1 (11%) reported ‘no’ associations. Of the four studies reporting on associations of major life stress and symptom exacerbation, the strength of association was ‘moderate’ for 2 (50%) studies and ‘weak’ and ‘none’ for one study each (25%). The strength of association for recent life events was ‘moderate to strong’ for 2 (50%) studies, ‘weak’ for 1 (25%) and ‘none’ for 1 (25%) study. The strength of association in both studies looking at daily strains was ‘weak’. The strength of association for low and high mood and psychological stress was ‘weak’ in both studies, whilst a ‘strong’ association was found for hopelessness as a factor for exacerbating symptoms in CD. Strength of association for long-term stress in UC was found to be ‘none’.

#### Temporality

Strong support for this criterion was found as all included studies were prospective cohort studies, a design that ensures exposure will precede outcome. In addition, all studies provided evidence in support of temporality. There was one study by Gracie et al. [47] where both directions were measured; however, for the purpose of this study, only the data where the exposure precedes the outcome was included. Thus, temporality was satisfied in all studies.

#### Coherence

Associations between psychological stress and IBD symptoms have been documented across Europe, North and South America and Australia. There is evidence from different research teams using different methods which supports this criterion strongly. However, in this review only cohort studies were included and therefore, this criterion was no applicable.

#### Consistency

Findings of associations between psychological factors and symptom exacerbation have been established in other populations [57–60]. Accordingly, this criterion was satisfied for all the studies.

#### Plausibility

The exposures selected in this review (psychological factors) meet the criteria for plausibility of scientific credible mechanism for causality. For example, empirical evidence from animal studies suggests potentially causal mechanisms between depression and inflammation [61, 62]. Therefore, this criterion was met for all the studies.

#### Biological gradient

This criterion examines if the changes in disease (symptom) activity correspond to changes in exposure (length or intensity of exposure to psychological factors). All the included studies reported corresponding changes of disease activity symptom with changes in exposure; however, there was no clear information about dose response in the studies (apart from Mardini et al. [41]) and therefore this criterion is not applicable.

#### Experiment

Whilst none of the 19 studies deliver experimental evidence, a number of studies including RCTs looking at psychological therapies and their effect on disease activity in IBD support the plausibility of a causal relationship between psychological factors and symptom exacerbation [63–65]. However, for this review as with the coherence criterion above, the experiment criterion is not applicable.

#### Causal association

The causal association scores range from weak to moderate for all of the psychological factors.

## Discussion

This systematic review and meta-analysis examined the association between psychological factors and symptom exacerbation in IBD using the Bradford Hill criteria to further evaluate causation. To our knowledge, this is the first systematic review utilising the application of Bradford Hill criteria to examine the causation between psychological factors and symptom exacerbation in IBD. We assessed 19 cohort studies looking at several psychological factors and symptom exacerbation in IBD and conducted a meta-analysis on five of these suitable studies. Using the Bradford Hill criteria, we found a weak to moderate causal relationship between psychological factors and symptom exacerbation. We did not find a statistically significant relationship between the psychological factors of perceived stress, depression or anxiety with symptom exacerbation when meta-analysis was performed. Our findings are consistent with previous limited evidence from meta-analysis which overall found a null association between psychological factors and symptom exacerbation in IBD. For example, Alexakis et al. [30] meta-analysis found no association between depressive states and disease course in IBD. However, there remains inconsistent findings within this area with some individual studies finding an association between psychological factors and exacerbation of IBD symptoms [17–19], whilst others have not [20–22]. This was our rationale for utilising the Bradford Hill criteria to comprehensively evaluate the strength of causation between psychological factors and symptom relapse in IBD.

These criteria are the most typically applied framework to assess causality [66]. However, there is a debate as to whether all of Hill’s criteria are of the same value. Some suggest that temporality is the most important criterion because causality cannot be assessed if the risk factor is not a predecessor to the outcome, whilst others suggest that the most important criterion is the experiment due to experiment being the only place where cofounders can be controlled and therefore their influence on causality isolated [67]. Nonetheless, both views are correct and not mutually exclusive of each other, which highlights the complexity of causality and supports the principle that the threshold for declaring causality should be high [35].

Taking this into account, all the studies included in this review established temporal direction or temporality from psychological factors to symptom exacerbation, the factors necessary to conclude causation according to Hill [31]. In addition, the criteria of strength of association, consistency and plausibility were also met, whilst biological gradient, coherence and experiment were not applicable. The scores were either 3 or 4 across all the studies as per Degelman [36], which enabled us to conclude that there is weak to moderate evidence to support a causal relationship between psychological factors as a whole and symptom exacerbation. This finding is consistent with some of the narrative reviews that have looked at a portion of psychological factors in IBD [25, 27, 68].

What might be confusing to some is that the strength of association (the first Hill criteria) was variable across the different psychological factors and varied between weak, moderate and strong. This computation was important in order to assess and determine which specific psychological factors were more strongly associated with symptom exacerbation, or if there was any difference between CD and UC studies. The results showed that the strength of association was moderate to strong for less than 50% of the studies. However, for example, when looking in depression, the studies that had a moderate/strong strength of association with symptom exacerbation had an overall larger study population size when compared to the studies having weak associations. This could be a chance finding or it could be that larger studies may be more reliable.

Utilising the Bradford Hill criteria could be a confusing or less familiar framework for most clinicians which might result in scepticism, particularly due to this review offering a conclusion that is not fully consistent with previous similar reviews about the role of psychological factors in IBD symptom exacerbation [30]. Nonetheless, the Bradford Hill criteria are an emerging and valuable technique for identifying causality and can be a useful guidance when there is an inconsistency in evidence and is a framework that has been seen more often in studies assessing causality [38, 69–71]. In our review, the Bradford Hill criteria have been applied to each of the psychological factors examined in the studies and enabled us to draw conclusions for each of them separately.

Whilst all of the 19 eligible studies included in the review looked at psychological factors (10 different in total), each of the studies examined a small subsample of psychological factors and used different tools to measure these. For example, four out of the ten psychological factors had only one study evaluating them [18, 19, 41, 45]. Furthermore, those psychological factors that were evaluated by a larger number of studies (such as depression, perceived stress and anxiety) used different tools and measures for them as well as a variety of statistical analyses. Thus, all the above made grouping for meta-analysis, summarising and drawing a definite conclusion about their impact on symptom exacerbation difficult. However, looking at a wider range of psychological factors has allowed for an all-inclusive approach. Although all the psychological factors were recorded using a variety of tools, the shared element was that all psychological factors were self-reported by the participants and no factor was objectively assessed by other means such as biomarkers.

### Strengths and limitations

The strengths of this review are the comprehensive and thorough approach taken to this ambiguous area of IBD care. By using the Bradford Hill approach to determine causality and including prospective studies only it enabled the avoidance of limitations associated with retrospective design and therefore enabled a rigorous approach to identifying the relationship between psychological factors and symptom exacerbation.

There are several limitations to this review. These include the limitations of the primary studies identified within the review as well as the limitations specific to the systematic process. For example, some of the studies identified were of a lower quality. Some studies had not reported data that could be used in our meta-analysis or described the type of analysis or methods used. In addition, many of the studies measured a range of psychological factors as well as employing different measurement tools. This heterogeneity made the synthesis difficult and limited the meta-analysis to five studies.

The results of any systematic review depend on the quality of the available literature. Whilst all the included studies were rated of high quality according to CASP 2017, it is important to clarify that this rating was relating to the study design and does not reflect the position of the study time in the hierarchy of evidence. Despite the studies being of high quality according to CASP, when evaluating the methodological quality of the studies for this review, we identified several weaknesses within them. For example, same of the studies had a small sample size or short follow-up. Short follow-up or small sample in cohort studies might not be sufficient to see the ‘true’ effect of psychological factors on symptoms [70] which can explain the variability in findings between studies with different study samples and follow-up times.

This review included four databases as well as reference lists as part of the search process to reduce the likelihood of missing important studies; however, there is still a possibility that some studies were missed. Due to the review having strict inclusion criteria, many studies were excluded. For example, the review was limited to English language; therefore, relevant studies in other languages could have been missed in the evidence. However, the screening process of studies was transparent and independently verified by 2 researchers to ensure only the most relevant studies with high methodological quality were included in this review.

### Overall applicability of evidence

The results of this review are applicable to adults with Crohn’s disease and ulcerative colitis. Given the findings from this review, there are two important messages. Firstly, although the causal link between psychological factors and symptom exacerbation was weak to moderate using the Bradford Hill criteria, there is evidence of a relationship. This finding fits with extensive reports from clinicians and patients that there is a relationship between psychological factors and symptom exacerbation in IBD [10, 11], whilst the uncertainty of evidence remains it is important to provide psychological assessment and support for the IBD population. Secondly, the unclear relationship between psychological factors and symptom exacerbation in IBD warrants further investigation. The limited availability of data suitable to combine for meta-analysis in this area requires large-scale randomised controlled trials to enable definitive answers. Standardising the use of measurement tools for psychological and disease symptoms would also aid future research.

## Conclusions

This review found limited evidence to support a relationship between psychological factors and symptom exacerbation in IBD. Taking account of the weak to moderate relationship from the Bradford Hill application and finding no statistically significant relationship from the meta-analysis suggests that these findings should be interpreted with caution and further studies are warranted.

## Supplementary information

**Additional file 1.** CASP Results

## Abbreviations

IBD: Inflammatory bowel disease
CD: Crohn’s disease
UC: Ulcerative colitis
SCCAI: Simple Clinical Colitis Activity Index
CINAHL: Cumulative Index to Nursing and Allied Health Literature
MEDLINE: Medical Literature Analysis and Retrieval System Online
PsychINFO: Psychological Information
EMBASE: Excerpta Medica Database

## References

[1] Bitton A, Dobkin PL, Edwardes MD, Sewitch MJ, Meddings JB, Rawal S (2008). Predicting relapse in Crohn’s disease: a biopsychosocial model. Gut..

[2] Molodecky NA, Soon IS, Rabi DM, Ghali WA, Ferris M, Chernoff G (2012). Increasing incidence and prevalence of the inflammatory bowel diseases with time, based on systematic review. Gastroenterology..

[3] Ng SC, Shi HY, Hamidi N, Underwood FE, Tang W, Benchimol EI (2017). Worldwide incidence and prevalence of inflammatory bowel disease in the 21st century: a systematic review of population-based studies. Lancet..

[4] Baumgart DC, Carding SR (2007). Inflammatory bowel disease: cause and immunobiology. Lancet..

[5] Frolkis AD, Dykeman J, Negrón ME, de Bruyn J, Jette N, Fiest KM (2013). Risk of surgery for inflammatory bowel diseases has decreased over time: a systematic review and meta-analysis of population-based studies. Gastroenterology..

[6] Bannaga AS, Selinger CP (2015). Inflammatory bowel disease and anxiety: links, risks, and challenges faced. Clin Exp Gastroenterol.

[7] Lesage A, Hagege H, Tucat G, Gendre J (2011). Results of a national survey on quality of life in inflammatory bowel diseases. Clin Res Hepato Gas.

[8] Ghosh N, Premchand P (2015). A UK cost of care model for inflammatory bowel disease. Frontline gastroenterol.

[9] Graff LA, Walker JR, Lix L, Clara I, Rawsthorne P, Rogala L (2006). The relationship of inflammatory bowel disease type and activity to psychological functioning and quality of life. Clin Gastroenterol H.

[10] Murray C (1930). Psychogenic factors in the etiology of ulcerative colitis and bloody diarrhea. Am J Med Sci.

[11] Moser (1993). Inflammatory bowel disease: patient’s beliefs about the etiology of their disease-a controlled study. Psychosom Med.

[12] Hemingway H, Marmot M (1999). Psychosocial factors in the aetiology and prognosis of coronary heart disease: systematic review of prospective cohort studies. BMJ.

[13] Booth J, Connelly L, Lawrence M, Chalmers C, Joice S, Becker C (2015). Evidence of perceived psychosocial stress as a risk factor for stroke in adults: a meta-analysis. BMC Neurol.

[14] Matcham F, Ali S, Hotopf M, Chalder T (2015). Psychological correlates of fatigue in rheumatoid arthritis: a systematic review. Clin Psychol Rev.

[15] Gu H, Tang C, Yang Y (2012). Psychological stress, immune response, and atherosclerosis. Atherosclerosis.

[16] Pickup JC (2004). Inflammation and activated innate immunity in the pathogenesis of type 2 diabetes. Diabetes Care.

[17] Porcelli P, Leoci C, Guerra V, Taylor GJ, Bagby RM (1996). A longitudinal study of alexithymia and psychological distress in inflammatory bowel disease. J Psychosom Res.

[18] Bitton A, Sewitch MJ, Peppercorn MA, de B Edwardes MD, Shah S, Ransil B (2003). Psychosocial determinants of relapse in ulcerative colitis: a longitudinal study. Am J Gastroenterol.

[19] Bernstein CN, Singh S, Graff LA, Walker JR, Miller N, Cheang M (2010). A prospective population-based study of triggers of symptomatic flares in IBD. Am J Gastroenterol.

[20] North CS, Clouse RE, Spitznagel EL, Alpers DH. The relation of ulcerative colitis to psychiatric factors: a review of findings and methods. Am J Psychiatry. 1990. 10.1176/ajp.147.8.974 2197886

[21] Vidal A, Gómez-Gil E, Sans M, Portella MJ, Salamero M, Piqué JM (2006). Life events and inflammatory bowel disease relapse: a prospective study of patients enrolled in remission. Am J Gastroenterol.

[22] Mikocka-Walus AA, Turnbull DA, Moulding NT, Wilson IG, Holtmann GJ, Andrews JM (2008). Does psychological status influence clinical outcomes in patients with inflammatory bowel disease (IBD) and other chronic gastroenterological diseases: an observational cohort prospective study. BioPsychoSocial medicine.

[23] Schoultz M, Atherton I, Hubbard G, Watson AJ (2013). Assessment of causal link between psychological factors and symptom exacerbation in inflammatory bowel disease: a protocol for systematic review of prospective cohort studies. Systematic reviews.

[24] Garrett JW, Drossman DA (1990). Health status in inflammatory bowel disease: biological and behavioral considerations. Gastroenterol..

[25] Searle A, Bennett P (2001). Psychological factors and inflammatory bowel disease: a review of a decade of literature. Psychol Health Med.

[26] Maunder RG, Levenstein S (2008). The role of stress in the development and clinical course of inflammatory bowel disease: epidemiological evidence. Curr Mol Med.

[27] Sajadinejad MS, Asgari K, Molavi H, Kalantari M, Adibi P. Psychological issues in inflammatory bowel disease: an overview. Gastroenterol Res Pract. 2012;2012. 10.1155/2012/106502 PMC3388477 22778720

[28] Maunder RG (2005). Evidence that stress contributes to inflammatory bowel disease: evaluation, synthesis, and future directions. Inflamm Bowel Dis.

[29] Greene BR, Blanchard EB, Wan CK (1993). Long-term monitoring of psychosocial stress and symptomatology in inflammatory bowel disease. Behav Res Ther.

[30] Alexakis C, Kumar S, Saxena S, Pollok R (2017). Systematic review with meta-analysis: the impact of a depressive state on disease course in adult inflammatory bowel disease. Aliment Pharmacol Ther.

[31] Hill AB (1965). The environment and disease: association or causation?. Proc R Soc Med.

[32] McInnes MD, Moher D, Thombs BD, McGrath TA, Bossuyt PM, Clifford T (2018). Preferred reporting items for a systematic review and meta-analysis of diagnostic test accuracy studies: the PRISMA-DTA statement. JAMA.

[33] CASP U (2017). Critical Appraisal Skills Programme (CASP). Qualitative research checklist.

[34] University of York: Centre for Review and Dissemination. Centre for Review and Dissemination: Systematic Reviews:CRD's Guidance for Undertaking Reviews in Health Care: Centre for Reviews and Dissemination. 2009.

[35] Roffey DM, Wai EK, Bishop P, Kwon BK, Dagenais S (2010). Causal assessment of occupational sitting and low back pain: results of a systematic review. Spine J.

[36] Degelman ML, Herman KM (2017). Smoking and multiple sclerosis: a systematic review and meta-analysis using the Bradford Hill criteria for causation. Mult Scler Relat Disord.

[37] Henriksen M, Creaby MW, Lund H, Juhl C, Christensen R (2014). Is there a causal link between knee loading and knee osteoarthritis progression? A systematic review and meta-analysis of cohort studies and randomised trials. BMJ Open.

[38] Mente A, de Koning L, Shannon HS, Anand SS (2009). A systematic review of the evidence supporting a causal link between dietary factors and coronary heart disease. Arch Intern Med.

[39] Barendregt JJ, Doi SA (2016). MetaXL user guide. Version.

[40] von Wietersheim J, Köhler T, Feiereis H (1992). Relapse-precipitating life events and feelings in patients with inflammatory bowel disease. Psychother Psychosom.

[41] Mardini HE, Kip KE, Wilson JW (2004). Crohn’s disease: a two-year prospective study of the association between psychological distress and disease activity. Dig Dis Sci.

[42] Kochar B, Barnes EL, Long MD, Cushing KC, Galanko J, Martin CF (2018). Depression is associated with more aggressive inflammatory bowel disease. Am J Gastroenterol.

[43] Bernstein MT, Targownik LE, Sexton KA, Graff LA, Miller N, Walker JR. Assessing the relationship between sources of stress and symptom changes among persons with IBD over time: a prospective study. Canadian Journal of Gastroenterology and Hepatology. 2016;2016. 10.1155/2016/1681507 PMC5067325 27795954

[44] Mikocka-Walus A, Pittet V, Rossel J, von Känel R, Anderegg C, Bauerfeind P (2016). Symptoms of depression and anxiety are independently associated with clinical recurrence of inflammatory bowel disease. Clin Gastroenterol Hepatol.

[45] Langhorst J, Hofstetter A, Wolfe F, Häuser W (2013). Short-term stress, but not mucosal healing nor depression was predictive for the risk of relapse in patients with ulcerative colitis: a prospective 12-month follow-up study. Inflamm Bowel Dis.

[46] Camara RJ, Schoepfer AM, Pittet V, Begré S, von Känel R (2011). Swiss Inflammatory Bowel Disease Cohort Study (SIBDCS) Group. Mood and nonmood components of perceived stress and exacerbation of Crohn’s disease. Inflamm Bowel Dis.

[47] Gracie DJ, Guthrie EA, Hamlin PJ, Ford AC. Bi-directionality of brain–gut interactions in patients with inflammatory bowel disease. Gastroenterology 2018;154(6):1635-1646. e3. 10.1053/j.gastro.2018.01.027 29366841

[48] North CS, Alpers DH, Helzer JE, Spitznagel EL, Clouse RE (1991). Do life events or depression exacerbate inflammatory bowel disease? A prospective study. Ann Intern Med.

[49] Mittermaier C, Dejaco C, Waldhoer T, Oefferlbauer-Ernst A, Miehsler W, Beier M (2004). Impact of depressive mood on relapse in patients with inflammatory bowel disease: a prospective 18-month follow-up study. Psychosom Med.

[50] Levenstein S, Prantera C, Varvo V, Scribano ML, Andreoli A, Luzi C (2000). Stress and exacerbation in ulcerative colitis: a prospective study of patients enrolled in remission. Am J Gastroenterol.

[51] Gaines LS, Slaughter JC, Horst SN, Schwartz DA, Beaulieu DB, Haman KL (2016). Association between affective-cognitive symptoms of depression and exacerbation of Crohn’s disease. Am J Gastroenterol.

[52] Flávia D’Agosto Vidal de Lima, da Rocha Ribeiro, Tarsila Campanha, Chebli LA, de Lima Pace, Fábio Heleno, de Miranda Chaves, Leonardo Duque, Ribeiro MS, et al. Mood swings in patients with Crohn’s disease: incidence and associated factors. Revista da Aockssociação Médica Brasileira (English Edition) 2012;58(4):481-488. 22930029

[53] Duffy LC, Zielezny MA, Marshall JR, Weiser MM, Phillips JF, Byers TE (1992). Comparison of stress indices in gauging clinical activity in patients with inflammatory bowel disease. J Trauma Stress.

[54] Duffy LC, Zielezny MA, Marshall JR, Weiser MM, Phillips JF, Byers TE, et al. Lag time between stress events and risk of recurrent episodes of inflammatory bowel disease. Epidemiology. 1991:141–5. 10.1097/00001648-199103000-00009 1932312

[55] Kanner AD, Coyne JC, Schaefer C, Lazarus RS (1981). Comparison of two modes of stress measurement: daily hassles and uplifts versus major life events. J Behav Med.

[56] Thoits PA. Dimensions of life events that influence psychological distress: an evaluation and synthesis of the literature. Psychosocial stress: Elsevier; 1983. p. 33-103.

[57] Quint JK, Baghai-Ravary R, Donaldson GC, Wedzicha JA (2008). Relationship between depression and exacerbations in COPD. Eur Respir J.

[58] Coventry PA, Gemmell I, Todd CJ (2011). Psychosocial risk factors for hospital readmission in COPD patients on early discharge services: a cohort study. BMC pulmonary medicine.

[59] Lokker ME, Gwyther L, Riley JP, van Zuylen L, van der Heide A, Harding R (2016). The prevalence and associated distress of physical and psychological symptoms in patients with advanced heart failure attending a South African medical center. J Cardiovasc Nurs.

[60] Oland AA, Booster GD, Bender BG (2017). Psychological and lifestyle risk factors for asthma exacerbations and morbidity in children. World Allergy Organization Journal.

[61] O’Malley D, Quigley EM, Dinan TG, Cryan JF (2011). Do interactions between stress and immune responses lead to symptom exacerbations in irritable bowel syndrome?. Brain Behav Immun.

[62] Ghia JE, Blennerhassett P, Collins SM (2008). Impaired parasympathetic function increases susceptibility to inflammatory bowel disease in a mouse model of depression. J Clin Invest.

[63] Triantafillidis JK, Merikas E, Gikas A (2013). Psychological factors and stress in inflammatory bowel disease. Expert Rev Gastroent.

[64] Ballou S, Keefer L (2017). Psychological interventions for irritable bowel syndrome and inflammatory bowel diseases. Clin Transl Gastroenterol.

[65] Gracie DJ, Irvine AJ, Sood R, Mikocka-Walus A, Hamlin PJ, Ford AC (2017). Effect of psychological therapy on disease activity, psychological comorbidity, and quality of life in inflammatory bowel disease: a systematic review and meta-analysis. Lancet Gastroenterol Hepatol.

[66] Ward A (2009). Causal criteria and the problem of complex causation. Med Health Care Philos.

[67] Kundi M (2006). Causality and the interpretation of epidemiologic evidence. Environ Health Perspect.

[68] Mikocka-Walus AA, Turnbull DA, Moulding NT, Wilson IG, Andrews JM, Holtmann GJ (2007). Controversies surrounding the comorbidity of depression and anxiety in inflammatory bowel disease patients: a literature review. Inflamm Bowel Dis.

[69] Rothman KJ, Greenland S (2005). Causation and causal inference in epidemiology. Am J Public Health.

[70] Kwon B, Roffey D, Bishop P, Dagenais S, Wai E (2011). Systematic review: occupational physical activity and low back pain. Occup Med.

[71] Talmage J (2010). So why does my back hurt doc?. Spine J.

