# Supplementary material for: Assessment of causal link between psychological factors and symptom exacerbation in inflammatory bowel disease: a systematic review utilising Bradford Hill criteria and meta-analysis of prospective cohort studies
Source: Syst Rev. 2020 Aug 1;9:169. doi: 10.1186/s13643-020-01426-2 (PMC7395978; doi:10.1186/s13643-020-01426-2)
Supplement: Supplementary file 1 — Additional file 1. CASP Results [file 13643_2020_1426_MOESM1_ESM.docx]

Additional File/ CASP Results

| **AUTHOR YEAR** | **1. Did the study address a clearly focused issue?** | **2. Was the cohort recruited in an**  **acceptable way?** | **3. Was the exposure accurately measured to minimise bias?** | **4. Was the outcome accurately measured to minimise bias?** | **5a.**  **Have the**  **authors identified all important confounding factors?** | **5b. Have they taken account of the confounding factors in the design and/or analysis?** | **6a. Was the follow up of subjects complete enough?** | **6b. Was the follow up of subjects long enough?** | **7. What are the results of this study?** | **8. Are results precise?** | **9. Do you believe the results?** | **10. Can the results be applied to the local population?** | **11. Do the results of this study fit with other available evidence?** | **12. What are the implications of this study for practice?** | **Bias Risk**  **Risk** |
| --- | --- | --- | --- | --- | --- | --- | --- | --- | --- | --- | --- | --- | --- | --- | --- |
| Bernstein 2010 | Yes | Yes | Yes | Yes | Yes | Yes | Yes | Yes | High-perceived stress was associated with an increased risk of flare up | Yes | Yes | Yes | Yes | Psychological factors contribute to IBD symptom flares | L |
| Duffy  1992 | Yes | Yes | Yes | Yes | Yes | Yes | Yes | Yes | Stress measures were moderately correlated correlation to disease | Yes | Yes | Yes | Yes | Influencing changes in stress perceptions may be useful in the treatment and prevention of IBD | L |
| Mardini 2004 | Yes | Yes | Yes | Yes | Yes | Yes | Yes | Yes | Levels of depressive symptoms are positively associated with future changes in CDAI | Don’t know | Don’t know | Yes | Yes | Routine psychological assessment may help to identify patients at higher risk for exacerbation | L |
| North  1991 | Yes | Yes | Yes | Yes | Yes | Yes | Yes | Yes | Life events were not associated with changes in GI symptoms | Don’t know | Don’t know | No | yes | Be cautious in attributing any special etiologic importance of life events or depressive symptoms | L |
| Mikocka-Walus  2008 | Yes | Yes | Yes | Yes | Yes | Yes | Yes | Yes | No significant relationship was found between psychological problems | Yes | Yes | Yes | Yes | Longer lasting studies with  larger and more homogenous groups of patients are needed. | L |
| Cohen 2014 | Yes | Yes | Yes | Yes | Yes | Yes | Yes | Yes | Fatigue’s association with all scores remained highly significant despite controlling for disease activity | Yes | Yes | Yes | Yes | Monitoring fatigue is a simple way to screen for overall disruption in patients with UC and CD. | L |
| Mittermaier 2004 | Yes | Yes | Yes | Yes | Yes | Yes | Yes | Yes | Depression and anxiety scores at baseline correlated with more frequent relapses during follow-up | may be a risk factor | yes | Yes | Yes | Assessment and management of psychological distress should be included in clinical treatment of patients with IBD | L |
| Duffy  1991 | Yes | Yes | Yes | No | Yes | Yes | Yes | Yes | Data indicated a strong association between stress exposures and new episodes of disease | Yes | Yes | Yes | Yes | The results underscore the importance of monitoring and managing stress in this group | L |
| Vidal  2006 | Yes | Yes | Yes | Yes | Yes | Yes | Yes | Yes | The number of life events was not associated with the rate of relapse | Yes | Yes | Yes | Yes | stressful life events do not trigger exacerbations in patients suffering from IBD | L |
| Greene 1993 | Yes | Don’t know | Don’t know | Don’t know | Don’t know | No | Yes | Yes | Daily stress appears to positively associate with daily IBD severity | No | No | No | Yes | Future work would help refine the stress-IBD model | H |
| Vidal De Lima  2012 | Yes | Yes | Yes | Yes | Yes | Yes | Yes | Yes | A high mood swing incidence (58%) was found in patients with CD. | Don’t know | Yes | Don’t know | Yes | Psychological assessment may be useful to detect and manage mood swings in patients with CD | L |
| Bitton  2007 | Yes | Yes | Yes | Yes | Yes | Yes | Yes | Yes | The interaction between perceived stress and avoidance coping were predictors of earlier relapse | Yes | Yes | Yes | Yes | Supports the importance of the biopsychosocial approach in identifying patients at high risk of relapse in CD | L |
| Bitton  2003 | Yes | Yes | Yes | Yes | Yes | Yes | Yes | Yes | A weak association between number of stressful events in the preceding month and time to relapse. This association strengthened in multivariate analysis | Yes | Yes | Yes | Yes | Interventions to reduce stress and improve coping may have a beneficial effect on health-related quality of life and coping skills in patients with IBD | L |
| von Wietersham  1992 | Yes | Don’t know | Don’t know | Yes | No | No | No | Yes | No significant relationship was found between psychological problems | Don’t know | No | No | No | No | H |
| Duffy  1991 | Yes | Yes | Yes | Yes | Yes | Yes | Yes | Yes | Stress exposed subjects had an increased risk of clinical episodes of disease | Don’t know | Yes | Yes | Yes | The relationship between stress and disease activity is perhaps best described as self-perpetuating | L |
| Langhorst 2013 | Yes | Yes | Yes | Yes | Yes | Yes | Yes | Yes | Short-term stress at the last visit before relapse were predictive for a relapse | Yes | Yes | Yes | Yes | Short-term stress might be predictive for relapse in patients with UC in clinical remission | L |
| Levenstein 2000 | Yes | Yes | Yes | Yes | Yes | Yes | Yes | Yes | Perceived Stress significantly increased the actual risk of exacerbation | Yes | Yes | Yes | Yes | Reinforce the utility of the biopsychosocial model in understanding the origin and course of disease | L |
| Camara 2011 | Yes | Yes | Yes | Yes | Yes | Yes | Yes | Yes | An association between perceived stress and exacerbation of CD | Yes | Yes | Yes | Yes | Future interventional studies should evaluate the treatment of anxiety and depression in CD | L |
| Garrett 1990 | No | Yes | Yes | Yes | No | Don’t know | Don’t know | No | For some individuals with Crohn's disease, daily stress is related to self-reported indicators of the illness | No | No | No | Yes | Health professionals should develop interventions to help patients develop more effective strategies | H |
| Graff 2006 | Yes | Yes | Yes | Yes | Yes | No | No | Yes | Those with active disease had higher levels of distress, health anxiety, and perceived stress, well-being and mastery, and poorer disease-specific QOL, relative to those with inactive disease. | Don’t know | Don’t know | Yes | Yes | It is important to be aware of related difficulties in patients with active IBD. There is a continued impact on QOL by the disease, even when it is inactive | L |
| Riley  1990 | Yes | Yes | Yes | Yes | No | No | No | Yes | Anxiety and depression ratings were similar in relapsers and non-relapsers | Don’t know | No | Don’t know | Don’t know | Seasonal factors may contribute to both onset and relapse of ulcerative colitis | L |
| Kochar  2018 | Yes | Yes | Yes | Yes | Yes | Yes | Yes | Yes | IBD patients with baseline depression were at an increased risk for relapse, surgery, or hospitalization | Yes | Yes | Yes | Yes | Providers should consider administering the PHQ-8 (depression measure) to capture those at greater risk for aggressive disease | L |
